# Short-term effect on pain and function of neurophysiological education and sensorimotor retraining compared to usual physiotherapy in patients with chronic or recurrent non-specific low back pain, a pilot randomized controlled trial

**DOI:** 10.1186/s12891-015-0533-2

**Published:** 2015-04-10

**Authors:** Philipp Wälti, Jan Kool, Hannu Luomajoki

**Affiliations:** Zurich University of Applied Sciences, School of Health Professions, Institute of Physiotherapy, Winterthur, Switzerland; Physiotherapie am Kohlplatz, Heiden, Switzerland; Rehabilitation Centre, Valens, Switzerland

**Keywords:** Chronic low back pain, Patient education, Sensory motor training, Physiotherapy, Randomized controlled trial

## Abstract

**Background:**

Non-specific chronic low back pain (NSCLBP) is a major health problem. Identification of subgroups and appropriate treatment regimen was proposed as a key priority by the Cochrane Back Review Group. We developed a multimodal treatment (MMT) for patients with moderate to severe disability and medium risk of poor outcome. MMT includes *a)* neurophysiological education on the perception of pain to decrease self-limitation due to catastrophizing believes about the nature of NSCLBP, *b)* sensory training of the lower trunk because these patients predominantly show poor sensory acuity of the trunk, and *c)* motor training to regain definite movement control of the trunk.

A pilot study was conducted to investigate the feasibility of MMT, prior to a larger RCT, with focus on patients’ adherence and the evaluation of short-term effects on pain and disability of MMT when compared to usual physiotherapy.

**Method:**

We conducted a randomised controlled trial (RCT) in a primary care physiotherapy centre in Switzerland. Outcome assessment was 12 weeks after baseline. Patients with NSCLBP, considerable disability (five or more points on the Roland and Morris Disability Questionnaire (RMDQ) and medium or high risk of poor outcome on the Keele Start Back Tool (KSBT) were randomly allocated to either MMT or usual physiotherapy treatment (UPT) by an independent research assistant. Treatment included up to 16 sessions over 8 to 12 weeks. Both groups were given additional home training of 10 to 30 minutes to be performed five times per week. Adherence to treatment was evaluated in order to assess the feasibility of the treatment. Assessments were conducted by an independent blinded person. The primary outcome was pain (NRS 0-10) and the secondary outcome was disability (RMDQ). Between-group effects with Student’s t-test or the Mann-Whitney U test and the standardized mean difference of the primary outcome were calculated.

**Results:**

Twenty-eight patients (46% male, mean age 41.5 years (SD 10.6)) were randomized to MMT (n = 14) or UPT (n = 14). Patients’ adherence to treatment was >80% in both groups. Pain reduction (NRS; [95% CI]) was 2.14 [1.0 to 3.5] in the MMT and 0.69 [-2.0 to 2.5.] in the UPT. The between-group difference was 1.45 [0.0 to 4.0] (p = 0.03), representing a moderate effect size of 0.66 [-0.1 to 1.5]. Reduction in disability on the RMDQ was 6.71 [4.2 to 9.3] in MMT and 4.69 [1.9 to 7.4] in UPT, with a non-significant between-group difference of 2.02 [-1.5 to 5.6] (p = 0.25). The required sample size for a RCT with six months follow-up was estimated at 170 patients.

**Conclusions:**

MMT was found to be feasible and to significantly reduce pain in the short term when compared with UPT. A future RCT with a six-month follow-up would require approximately 170 patients.

**Trial registration:**

Current Controlled Trials ISRCTN66262199. Registered 8 January 2014.

**Electronic supplementary material:**

The online version of this article (doi:10.1186/s12891-015-0533-2) contains supplementary material, which is available to authorized users.

## Background

Low back pain (LBP) is a major international health problem with a lifetime prevalence of 80–85% [1]. In Switzerland, low back pain generates direct medical costs of 3.4 billion Euros, corresponding to 6.7% of total Swiss health care expenditures, and 8.4 billion Euros of total costs per year [2]. A specific diagnosis of LBP, such as nerve root compression, spinal stenosis and definite instability, is only present in about 15% of cases. In the remaining 85% of patients, LBP is non-specific [3]. Research on subgroups of LBP and the evaluation of tailored treatment regimens has been declared as one of the most important future fields of research [4]. Some studies, with specific treatments addressed at defined subgroups, showed better results than others, where a “one size fits all” treatment was used [5,6].

Various research groups have demonstrated the potential value of focusing on abnormal cortical processing of the central nervous system (CNS) in patients with non-specific chronic low back pain (NSCLBP) [7-14]: abnormal cortical processing includes *cognitive*, *sensory* and *motor* disturbances.

One trial, in which neurophysiological education was applied to patients with NSCLBP, showed a significant change (p < 0.05) in catastrophizing beliefs about pain or injury and in inappropriate coping strategies, measured with the pain catastrophizing scale (PCS) [10]. This indicates that a *cognitive* approach may play an important role in the treatment of NSCLBP and explains why our multimodal treatment (MMT) includes education on the neurophysiology of pain.

Flor et al. applied two-point-discrimination (TPD), or graphaesthesia-training, on patients with phantom limb pain (amputees). This led to a significant reversal in abnormal cortical processing, shown by functional magnetic resonance imaging (fMRI), and a reduction in phantom limb pain (p = 0.002), positively associated with improved sensory discrimination ability [13]. This effect has not yet been seen in patients with NSCLBP. Nevertheless, research has shown exceeded patterns of activation in the primary somatosensory cortex, elicited through the application of subcutaneous electric stimuli to the lower back, which correlated with the severity of chronicity in patients with NSCLBP (r = 0.74) [8]. This suggests that *central sensory changes* play a role in NSCLBP and that TPD training may reverse it. This is the reason the decision was made to integrate TPD training into MMT.

Processing in the *motor* cortex was extensively exceeded in patients with phantom limb pain, shown on fMRI. It was reversed (less diffuse, more confined) after six sessions of imagined movement and showed significant pain reduction (p = 0.0005) [14]. Moseley et al. found distorted body image and tactile function in patients with LBP [11], plus delayed recognition of right or left hand in patients suffering from chronic regional pain syndrome (CRPS) [15]. Delayed activation of the deep trunk-stabilizing muscles in motor tasks [16,17] and poor movement control of the lumbar spine [18,19] are other motor aspects associated with NSCLBP. These impairments are thought to be caused by abnormal processing in the *motor* cortex. MMT therefore includes exercises on imagined movement, selective activation of deep trunk muscles and recognition training using pictures of the trunk at different angles.

To our knowledge, a multimodal approach including patient education and sensorimotor retraining has been described only in a small exploratory study (n = 3) [20]. Randomized controlled trials (RCT) with a longer term follow-up are required to evaluate the effect of MMT. However, since the feasibility of MMT is currently unknown, it is valuable to evaluate the short-term effects in a pilot study, prior to conducting a larger RCT with a longer term follow-up. For the evaluation of the theoretical basis of this intervention, the development and the implementation of the multimodal treatment into a study design where the effect of the programme and also the feasibility of the study process can be assessed, we draw valuable advice from the MRC framework for the development of complex interventions [21].

### Objective

This pilot study was designed to investigate the short-term effects of multimodal treatment on pain and disability in a clinical subgroup of patients with NSCLBP. Adherence to treatment was evaluated to provide an assessment of the treatment feasibility. Results were used to estimate the required sample size for a larger RCT to evaluate longer term effects.

## Method

### Study design

This was a single-centre, assessor-blinded RCT carried out at the *medbase* centre for health care in Saint Gallen, Switzerland. Assessments were performed at baseline before first treatment and 12 weeks after baseline. Following inclusion, written informed consent and baseline assessment, patients with NSCLBP were randomly allocated to either the multimodal treatment group (MMT) or usual physiotherapy treatment group (UPT). Randomization of the pre-defined number of 28 patients was concealed, using a randomization list with a block size of four, which was generated electronically before start of the trial. Patients were treated by two independent physiotherapists, one in the MMT group and the other in the UPT group. The follow up assessments were conducted by an independent physiotherapist blinded to the group assignment. Treatment was free of charge for all patients participating in the study. The trial was approved by the local ethics committee (ethics committee of the canton Saint Gallen, Switzerland. Ethics committee trial identification number: EKSG 12/149/1B).

### Recruitment

Telephone contact was made with referring general practitioners (GP), chiropractors and rheumatologists. Fifteen received a 20-minute power point presentation on the background of the trial and were supplied with flyers outlining the study. In addition, human resources managers of local companies were contacted and requested to distribute information to their employees. Patient recruitment was also achieved through advertisements in a local newspaper. Interested participants were referred to an online questionnaire for a first eligibility check. Suitable candidates were then contacted and invited for a final eligibility check, informed consent and baseline assessment.

### Study population

The inclusion criteria were designed to recruit patients with moderate to severe disability and pain and were in accordance with the definition in the European guidelines for the management of NSCLBP [4]. Eligible were: men and women aged 18 to 60 years; a LBP history of three months or more; at least moderate disability, i.e. five points or more on the RMDQ [22,23]; and medium or high risk of poor outcome, as evaluated with the Keele Start Back Tool (KSBT) [24]. Participants were excluded when they had nerve root pain, a diagnosed specific spinal pathology (such as malignancy, fracture, infection, or inflammatory joint or bone disease), were pregnant or less than 6 months postpartum, had a coexisting major medical disease causing a relative or absolute contraindication to general exercise, had undergone spinal surgery within the preceding two years, or had had an intra-articular or perineural steroid injection on the lumbar spine during the previous five months. Participants had to be able to speak and read German, have a person available to assist them with home training, have access to the internet and consent to a time expenditure of 30 minutes, five times per week for eight weeks, to perform a home programme and one/two 30-minute physiotherapy sessions per week.

### Treatment dosage

Physiotherapy treatment dosage in both groups was one or two sessions per week during the 8 week programme, with a maximum of 16 sessions. Patients were given home assignments to be performed for 30 minutes five days per week. An extension to a maximum of twelve weeks was allowed to account for discontinuity, such as holiday or other time issues.

### Home training

Patients from both groups had personalized access to a web-based home training interface (HTI) to guide the home assignments. The HTI recorded adherence to assignments and allowed evaluation of performance (further details outlined in the treatment paragraphs below).

### Multimodal treatment group

MMT included: 1. Education on the neurophysiology of pain; 2. Sensory retraining; and 3. Motor retraining. Treatment was aimed at reducing pain and disability and, potentially, addressing associated abnormal cortical processing in NSCLBP. Additional file 1 provides a detailed time schedule of each part of the MMT.

**Figure 1:**
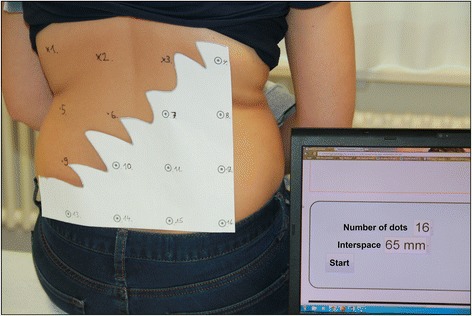
**Sensory Retraining Tool on the home training Interface for the multimodal treatment group.** Example of a gauge plotted on patient’s back. The specifications of the gauge (16 dots, 65 millimetres inter-punctual distance) were imputed on the SRT as visible on laptop-screen, SRT ready to start.

1. *Patient education* on the neurophysiology of pain was aimed at the reduction of patients’ perception of pain and disability, a reconsideration of protective behaviour and self-limitation resulting from fear of movement, assistance in regaining a confident and positive perspective of their abilities and acknowledgment of the beneficial value of activity. The content of this section was based on a RCT by Moseley et al. that demonstrated a beneficial effect of intensive neurophysiological education in patients with NSCLBP [11]. Our education began with an outline of a bio-psychosocial model of LBP, including cortical dysfunction in pain and body perception. Emphasis was placed on how this model might explain the features of the participants’ LBP experiences. Between two and four education sessions were held. Patients received a copy of the book “Explain Pain” (German translation) [25]. During the first two weeks, patients were required to read ten pages of the book each day and, using the HTI, answer 18 relevant questions on each section.
2. The aim of the *sensory retraining* section was to restore discriminate sensory acuity of the lower back and, potentially, to restore normal cortical processing in the sensory cortex, as Flor et al. have shown in patients with phantom limb pain [26]. Depending on the patients’ baseline lower back TPD-threshold, each received a set of gauges (like tracing papers) with 16 to 20 numbered dots with equal inter-punctual distance. Patients then had to transfer their appropriate gauge inter-punctual value to a Sensory Retraining Tool (SRT) integrated into their HTI (Figure 1). Each patient had an aid person to hold the gauge onto the patient’s back, to plot the dots through the paper, to number them and press the start button on the SRT. The SRT displayed dot numbers in randomized order and the aid had to press the relevant dot onto the patient’s back with a pencil, so that the patient could discern and identify the number of the dot being stimulated (Figure 2). The answer was confirmed with true or false on the SRT. When performance reached 80% correct answers, treatment progressed to the next gauge with an inter-punctual distance of five millimetres smaller. Likewise, letters and three-letter words were written directly onto the back. Tactile acuity was shown to be reduced in patients with LBP and correlated with movement control impairment [27]. A similar treatment was described by Wand et al. in a multi-baseline study reducing pain and disability in patients with NSCLBP [20].

**Figure 2:**
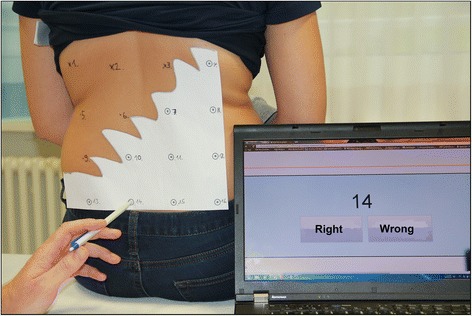
**Sensory Retraining Tool on the home training Interface for the multimodal treatment group.** SRT started: The patient has to discern the pressed dot number 14. The aid person then confirms the answer with right or wrong on the SRT and the next number appears on the screen, and so on.
3. The goal of the *motor retraining* was to improve movement control of the lower back [28]. Patients performed laterality recognition training. They were shown photographs of a human trunk rotated or side-bent to right or left. Patients had to determine the perspective of the picture as quickly as possible, using the computer programme Recognise® [29] linked to the HTI. In a next step, patients observed videos showing movements of increasing difficulty. The movements then had to be performed either physically or mentally as motor imagery exercise, depending on the patient’s pain intensity and ability. The first level of difficulty included small lumbar movements, such as pelvic tilting and slight side-bending. The second level involved larger unidirectional and combined lumbar movements. Movement patterns which provoked LBP were individually identified. Motor retraining exercises were instructed using mirrors, smartphone photographs and the patient’s hands, as a reminder of the experiences and to improve positive movement. The aim was to enable patients to transfer their acquired knowledge to any situation in which their LBP arose, to modulate and improve their movement behaviour and to reduce pain.

### UPT group

**Figure 3:**
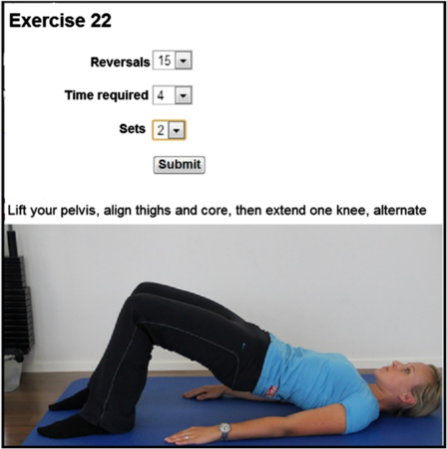
**Home training Interface of the control group.** For each exercise and time performed at home, these parameters had to be imputed on the HTI by the patient as shown on the picture: Number of reversals; number of sets; time required.

At the initial visit, patients were given basic patient education on adequate behaviour when having an exacerbation of LBP: a short period of protection followed, as soon as possible, by a return to normal movement, work and leisure activities. Further sessions addressed signs and symptoms. Two-thirds (i.e. 20 minutes) of each session consisted of active treatment, such as exercises for strengthening muscles, neuro-meningeal mobilisation and stretching muscles. A maximum of ten minutes of passive applications per session was allowed (such as massage, manual therapy, electrotherapy, mud packs). These activities are in accordance with the European guidelines for the management of active rehabilitation [4]. Each patient received instruction on the HTI to perform an individual home exercise programme. To observe adherence to the home programme, patients had to report their routine of performing exercises on the HTI (Figure 3).

### Outcomes

Feasibility-related outcomes were: suitability of recruitment; patients’ ability to complete each part of the home training programme; suitability of the time frame set; treatment attendance; and adherence to home assignments.

The primary outcome was mean pain intensity over the prior 7 days (NRS 0-10). Secondary outcomes were disability, measured with the Roland and Morris Disability Questionnaire (RMDQ), and patient-specific disability, measured with the patient-specific functional scale (PSFS), an outcome measure found to be reliable and responsive in patients with NSCLBP [30]. Fear avoidance beliefs were measured using the fear avoidance beliefs questionnaire FABQ [31,32] and catastrophizing thoughts with the pain catastrophizing scale PCS [33,34]. Movement control impairment (MCI) was measured by means of six movement control tests, with established validity and reliability [18,19], and sensory acuity of the lower back by measuring the TPD threshold [35]. Sick leave and analgesic intake during the last seven days were recorded at both assessments.

### Statistics

Treatment feasibility was defined as 80% patient participation over the complete programme (i.e. 8 to 12 weeks) and completion of at least 80% of each part of the home training assignments. For MMT, we aimed at ≥ 80% correct answers to the questions related to the lessons in the book “Explain Pain”; ≥ 80% of the required quota of 10 × 60 images on recognition training using the Recognise® software; ≥ 80% completion of the required quota of 55 sets of sensory training with the SRT. Time and frequency spent on the movement control retraining exercises were recorded. For UPT, we aimed at ≥ 80% compliance with the 40 home training assignments. Satisfaction with treatment was evaluated in both groups. Patients were asked to rate whether the contents of each section were helpful using a five point Likert scale, ranging from “strongly agree” to “strongly disagree”.

Outcome analysis was by “intention to treat”. The results of patients fulfilling the before-mentioned requirements for treatment adherence were analysed in the per protocol analysis.

We compared changes within and between groups for the primary and secondary outcomes. We used a two-tailed Student’s t-test for data showing normal distribution, which was tested with the SHAPIRO-Wilks test, and a two-tailed Mann Whitney U test where data were not normally distributed. Results were analysed with SPSS 20. Confidence intervals for the median difference (e.g. for the Mann Whitney U test) were calculated with the software r, version 3.1.1 using the Hodges-Lehman test.

## Results

### Study population

**Figure 4:**
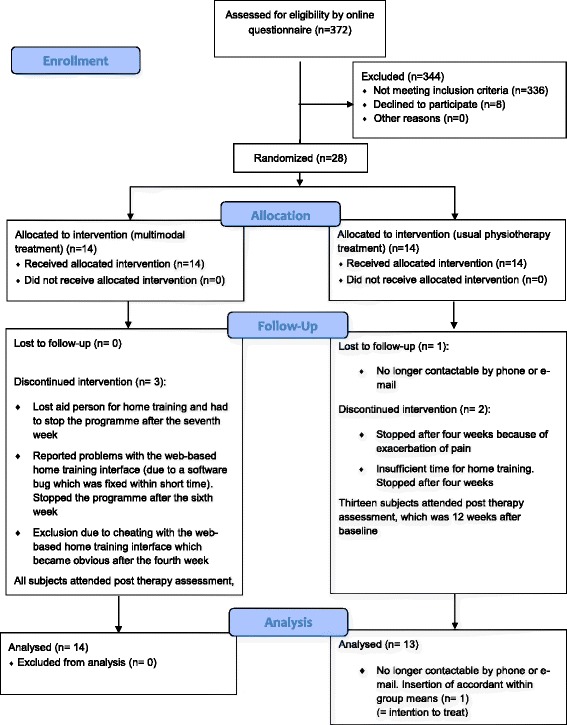
**Patient flow diagram.** CONSORT 2010 Flow Diagram.

Figure 4 shows the participant flow. All baseline characteristics, except gender and pain catastrophizing (PCS), were comparable (Table 1). One patient in the UPT group dropped out (treatment attendance stopped and not contactable by phone or email). The missing data of this dropped out patient were ignored and not statistically substituted (as this would have narrowed the CI what would have increased the chance at type 1 error). All other outcome data were complete (MMT n = 14, UPT n = 13).

**Table 1:** **Study population, baseline characteristics**

	**MMT (n = 14)**	**UPT (n = 14)**
Age, mean (SD)	41.57 (9.77)	41.71 (12.21)
Gender: female (%)	9 (64.3%)	6 (42.9%)
Pain NRS, mean (SD)	4.86 (1.61)	4.64 (1.82)
Disability PSFS, mean (SD)	5.43 (1.58)	5.48 (1.25)
Disability RMDQ, mean (SD)	10.21 (4.44)	11.21 (3.95)
Fear avoidance beliefs FABQ, mean (SD)	23.93 (11.58)	25.92 (12.28)
Pain catastrophizing PCS, mean (SD)	14.43 (7.62)	20.08 (8.24)
Sensory acuity TPD, mean (SD)	70.71 (14.39)	70.71 (14.12)
Movement control, mean (SD) number of positive tests out of 6	2.64 (1.55)	2.50 (1.09)
Days of work lost during prior 7 days pre-therapy	None	None
Pain medication during prior 7 days		
- none	7	8
- NSAID	5	5
- opioids	2	1

MMT: multimodal treatment (group); UPT: usual physiotherapy treatment (group); SD standard deviation; NRS numeric rating scale; PSFS patient-specific functional scale; RMDQ Roland and Morris Disability Questionnaire; FABQ Fear Avoidance Beliefs Questionnaire; PCS Pain Catastrophizing Scale; TPD two-point-discrimination; NSAID non-steroidal anti-inflammatory drug.

### Feasibility

#### Recruitment

The time span required to recruit the 28 patients was seven months (October 2012 to May 2013). Inclusion criteria excluded 90% of all persons interested in study participation. Thirty-six of the 372 interested persons met the inclusion criteria. Twenty-eight gave written informed consent and were assigned either to the MMT (n = 14) or UPT (n = 14). Additional files 2 and 3 provide a detailed description of recruitment resources and the eligibility process.

#### Drop-out and missing data

Six patients (three each from the MMT and UPT groups) stopped programme attendance before completion of the eight weeks of participation for cogent individual reasons (Table 2). All patients, except one, in the UPT attended post-therapy outcome assessment (MMT n = 14, UPT n = 13).

**Table 2:** **Individual reasons for stopping programme participation**

**Multimodal treatment group**	**Usual physiotherapy treatment group**
1 no aid for home training (week 7)	1 exacerbation of pain (week 4)
1 problems with the home training interface (week 4)	1 no time for the home training (week 4)
1 inappropriate and misleading use of the home training interface (week 4)	1 stopped treatment, reason unknown (week 7)

#### Treatment frequency, time frame and home programme adherence (per protocol analysis)

A per protocol analysis (MMT n = 11, UPT n = 11) was conducted because inclusion of the six drop-out patients’ data would have confounded the outcomes. Treatment was found to be feasible. The pre-defined level of treatment adherence was reached: in each group more than 80% adhered to the minimum performance of ≥80% of each section of the assigned home programme (Table 3). Treatment frequency for MMT was mean 8.6 (SD 2.1) and for UPT 8.0 (SD 1.8) sessions. The programme was carried out within the pre-determined timeframe of eight to twelve weeks (mean MMT 9.6, UPT 9.9).

**Table 3:** **Home training assignments; patients’ adherence and performance**

**MMT assignment**	**MMT performance (patients participating on the complete MMT programme (n = 11))**
Neurophysiological education: answering 184 questions in ten home assignments	81% (9/11) answered >80% of the questions correctly
Recognise® Software: determination of perspective of 10 × 60 pictures of the back.	81% (9/11) determined >80% of the pictures correctly
Sensory retraining with 55 sets of stimuli (pressed points, letters and 3-letter-words)	81% (9/11) fulfilled >80%
Motor retraining exercises	81% (9/11) reported performing exercises five times a week
Motor retraining exercise performance per day	
Frequency/day (mean, SD)	2.44 (0.81)
Minutes/day (mean, SD)	11.68 (3.17)
**UPT assignment**	**Eleven patients participated on the complete UPT programme (n = 11)**
Individually assigned exercises, performance reported on the HTI	91% (10/11) reported performance five times a week

MMT multimodal treatment (group); UPT usual physiotherapy treatment (group).

Patients’ satisfaction with treatment was sufficient and comparable in both groups, although the time spent on sensory retraining in the MMT group was experienced as too long by some patients (see Additional files 4 and 5).

#### Treatment outcome

Table 4 shows that mean pain over the prior week decreased significantly more in the intervention group, with a between-group difference of 1.45 [95% CI 0.0 to 4.0] (p = 0.03), resulting in a moderate effect size of 0.66 [95% CI -0.1 to 1.5]. No significant difference between groups was found for disability (RMDQ and PSFS), fear avoidance beliefs (FABQ) or pain catastrophizing (PCS). Sensory acuity of the lower back (TPD threshold) showed a significant mean between-group difference in favour of MMT (p = 0.02). Movement control remained unchanged. Sick leave did not occur at any point.

**Table 4:** **Treatment outcomes**

	Within-group mean difference [95% CI] *		**Between-group mean difference [95% CI], (p-value 2-tailed)**
	**MMT n = 14**	**UPT n = 13**	
***Primary outcome***			
Pain NRS	2.14 [1.0 to 3.5]	0.69 [-2.0 to 2.5.]	1.45 [0.0 to 4.0] (p = 0.03)
***Secondary outcomes***			
Disability PSFS	2.55 [1.3 to 3.8]	1.13 [-0.1 to 2.4]	1.42 [-0.25 to 3.09] (p = 0.09)
Disability RMDQ	6.71 [4.2 to 9.3]	4.69 [1.9 to 7.4]	2.02 [-1.5 to 5.6] (p = 0.25)
Fear avoidance beliefs FABQ	4.79 [0.6 to 8.9]	3.85 [-2.8 to 10.5]	0.94 [-6.3 to 8.2] (p = 0.79)
Pain catastrophizing PCS	3.43 [0.1 to 6.7]	6.15 [0.2 to 12.1]	-2.73 [-9.3 to 3.8] (p = 0.40)
Sensory acuity TPD	11.79 [6.8 to 16.8]	-2.69 [-14.3 to 8.9]	14.48 [2.2 to 26.8] (p = 0.02)
Movement control, positive out of 6 tests	-0.29 [-2 to 1]	-0.69 [-1.5 to 0.0]	0.50 [-1.0 to 2.0] (p = 0.29)
Pain medication during prior 7 days	13	11	
- none		2	
- NSAID	1	0	
- opioids		1 missing data	

MMT multimodal treatment (group); UPT usual physiotherapy treatment (group); *[95% CI] 95 percent confidence interval for normal distributed data; p-value; NRS numeric rating scale; PSFS patient-specific functional scale; RMDQ Roland and Morris Disability Questionnaire; FABQ Fear Avoidance Beliefs Questionnaire; PCS Pain Catastrophizing Scale; TPD two-point-discrimination; NSAID non-steroidal anti-inflammatory drug.

## Discussion

### Feasibility

#### Recruitment and programme adherence

The recruitment rate was lower than expected, with 90% of interested persons excluded. However, the target group of patients with considerable pain (5 on the NRS) and disability (10 points on the RMDQ) could be recruited. The stringent admission criteria may have contributed to the good patient compliance rate and positive short-term outcome.

Programme adherence was similarly satisfying in both groups. Cogent individual reasons led to two withdrawals of participants in the MMT: in one case, the patient’s aid person withdrew; in the other case, the patient was irritated by the fact that the HTI had difficulty in running on his Samsung tablet (although the system was updated to run on an android device within four days, the patient had already withdrawn). Both patients attended post-therapy assessment. Inappropriate and misleading use of the home training on the HTI was observed in one patient in the multimodal treatment group, leading to termination of treatment. This person also attended post-therapy assessment. The time spent on the home programme was recorded by the HTI, which was considered more accurate and valid than self-reporting.

Cogent individual reasons also led to two withdrawals from the UPT: one person was forced to stop because of pain exacerbation and another person could not afford the time required for home training. We found no exact data indicating how many NSCLBP patients showed exacerbation of pain during usual physiotherapy care treatment. Only one participant had no time to exercise, indicating high adherence to our home programme compared with the 70% of non-engagement in given home exercises prescribed by physiotherapists to patients with CLBP, as reported in a systematic review [36]. Programme attendance in our study was consistent with other studies in the field of NSCLBP, showing attrition rates ranging from 10 – 42% [37-39]).

#### Treatment frequency and time frame

The mean number of 8.5 treatment sessions was below the assumed average of 12 sessions, and therefore well in line with usual prescriptions in NSCLBP. Unfortunately, statistical data on the number of physiotherapy sessions for patients with NSCLBP are not available for comparison. It was feasible to perform the treatment programme within 12 weeks, comparable with 10 weeks in the study by Wand et al. [20]).

#### Outcome

The 1.45 point between-group difference in pain was smaller than the minimally clinical important difference of 1.7, as stated in the IMMPACT recommendations by Dworkin et al. In the same publication, the IMMPACT recommendations suggest that the minimally clinical important difference between groups is smaller than that in an individual patient. Between-group changes on a 1 to 10 scale ranged from 0.9 to 1.5 in nine drug studies concerning pain relief in patients with knee osteoarthritis, reviewed by Dworkin et al. [40].

The negative outcome regarding pain catastrophizing (PCS) was unexpected; the more so since pain was significantly reduced in the MMT group. Moseley et al. showed a significant decrease in catastrophizing beliefs on the PCS after intensive neurophysiology education on pain in patients with NSCLBP [10]. Pain catastrophizing might be further reduced by prolongation of the education, treatment and home assignments.

TPD threshold in our patients was 70 mm (SD14), which is slightly higher than in a previous study in a population with NSCLBP that reported 61 mm (SD 13) [27]. Sensory acuity increased only in the MMT and is, therefore, likely to be the result of the sensory retraining.

Movement control decreased slightly in both groups, but more clearly in the UPT group. This might be explained by the fact that, due to restricted resources, baseline assessment and post-treatment assessment were performed by different assessors, with the post-treatment assessor rating more conservatively. In future research, baseline and post-treatment tests for MCI should be rated by an independent blinded assessor using video recordings.

#### Strengths

The study showed that this particular population of NSCLBP patients was able to participate in a multimodal treatment (intervention group) or a usual physiotherapy treatment (UPT group) and report their performed home training on a personal web interface. Our study shows that ≥ 80% of patients engaged in at least 80% of the requested home exercises, apart from those with cogent individual reasons for withdrawal from programme participation. This RCT including 28 patients demonstrates the pain reduction potential of the MMT.

#### Limitations

The outcomes of this study have to be interpreted with caution because of the small sample size, as there is evidence that the risk of a type 1 error is larger in small sample sizes [39,41]. We used a pre-defined convenient sample size of n = 28 on the assumption that this data would give us enough information to ascertain the feasibility of a larger RCT.

Differences between groups for disability on the RMDQ, as well as on the PSFS, were not significant. The small between-group difference in change on fear avoidance beliefs (FABQ) may be explained by low responsiveness of the FABQ identified in a review [42]. We chose to apply this measure nevertheless since it had been validated in NSCLBP-populations and found to be a reliable and commonly-used measure of cognitive aspects of pain and function [41]. Another limitation is that patients recruited by advertisement may be different from those referred by physicians. However, the same inclusion criteria were used for all patients and randomization divided patients from both recruitment sources equally over both treatment groups.

Our assumptions on changes to the central nervous system were based on studies with comparable, but not identical, diagnoses. Based on these assumptions, we designed a treatment targeting three topics: education, sensory system and motor system. The question of causative relationship between NSCLBP and changes in the central nervous system would require functional imaging to evaluate changes in the central nervous system.

#### Clinical applicability

To our knowledge, this is the first study in a NSCLBP population to use the internet for treatment and require an aid person for home exercises. These requirements reduce its general applicability in physiotherapy. Within the next ten years, a rapid increase in the use of technology is expected and technical solutions may be developed to enable independent exercise.

#### Sample size of a RCT to evaluate long term results

Our results allow cautious sample size estimation for a study with a six-month follow-up. In the present study, the true mean between-group difference was 1.45 and the pooled standard deviation 2.11. Based on the assumption of a smaller true mean between-group difference in favour of MMT of 1.0 NRS point and a SD of 2.2. at six months, a power of 0.8 and a Type I error probability of 0.05 and accounting for 10% drop-outs, a total of 170 patients would be needed (g power software version 3.1.7 [43]).

## Conclusion

Both treatment programmes were feasible. Patients in both treatment groups showed good adherence both to treatment sessions and home assignments. Although the size of the effect was moderate, multimodal treatment reduced pain compared with usual physiotherapy. The results must be interpreted with caution because of the small sample size. MMT focussed on cognitive aspects concerning pain, sensory acuity of the lower back, movement control and body-awareness in daily living.

The multimodal treatment approach seems to be a potential alternative to conventional physiotherapy treatment. Based on our results, we estimate that 170 patients are needed to evaluate the six months’ effects. To increase the recruitment rate from approximately 50 to 200 eligible participants per year the study will be conducted in four centres.

## Additional files

Additional file 1:
**Content of sessions and home assignments of multimodal treatment.**
Additional file 2:
**Recruitment sources.**
Additional file 3:
**Online eligibility testing.**
Additional file 4:
**MMT Patients satisfaction with treatment.**
Additional file 5:
**UPT Patients satisfaction with treatment.**

## Abbreviations

ES: Effect size
FABQ: Fear Avoidance Beliefs Questionnaire
fMRI: Functional magnetic resonance imaging
GP: General practitioner
HTI: Web-based home training interface
KSBT: Keele Start Back Tool
LBP: Low Back pain
MCI: Movement control impairment
MMT: Multimodal treatment (group)
NRS: Numeric rating scale (NRS)
NSAID: Non-steroidal anti-inflammatory drug
NSCLBP: Non-specific chronic low back pain
PCS: Pain Catastrophizing Scale
PSFS: Patient-Specific Functional Scale
RCT: Randomised controlled trial
RMDQ: Roland and Morris Disability Questionnaire
SD: Standard deviation
SRT: Sensory Retraining Tool
TPD: Two-point-discrimination
UPT: Usual physiotherapy treatment (group)
[95% CI]: 95% confidence intervals

## References

[1] WHO. The burden of musculoskeletal conditions at the start of the new millennium. World Health Organ Tech Rep Ser. 2003; 919, i-x, 1-218. 14679827

[2] Interpharma and Polynomics. Gesundheitsausgaben und Krankheitskosten. [http://www.interpharma.ch/sites/default/files/documents/polynomics-2011_gesundheitsausgaben-und-krankheitskosten_d.pdf]. Sept. 2011.

[3] Hestbeak L, Leboeuf-Yde C, Manniche C (2003). Low back pain: what is the long-term course? A review of studies of general patient populations. Eur Spine J.

[4] Airaksinen O, Brox J, Cedraschi C, Hildebrandt J, Klaber-Moffett J, Kovacs F (2004). European guidelines for the management of chronic non-specific low back pain.

[5] Billis E, McCarthy C, Oldham J (2007). Subclassification of low back pain: a cross-country comparison. Eur Spine J.

[6] Fersum KV, Dankaerts W, O'Sullivan PB, Maes J, Skouen JS, Bjordal JM (2010). Integration of sub-classification strategies in RCTs evaluating manual therapy treatment and exercise therapy for non-specific chronic low back pain (NSCLBP): a systematic review. Br J Sports Med.

[7] Flor H. Cortical reorganisation and chronic pain: implications for rehabilitation. J Rehabil Med. 2003;(41 Suppl):66-72. 10.1080/16501960310010179 12817660

[8] Flor H, Braun C, Elbert T, Birbaumer N (1997). Extensive reorganization of primary somatosensory cortex in chronic back pain patients. Neurosci Lett.

[9] Lotze M, Moseley GL (2007). Role of distorted body image in pain. Curr Rheumatol Rep.

[10] Moseley GL, Nicholas MK, Hodges PW (2004). A randomized controlled trial of intensive neurophysiology education in chronic low back pain. Clin J Pain.

[11] Moseley GL (2008). I can't find it! Distorted body image and tactile dysfunction in patients with chronic back pain. Pain.

[12] Wand BM, O'Connell NE (2008). Chronic non-specific low back pain - sub-groups or a single mechanism?. BMC Musculoskelet Disord.

[13] Flor H (2002). Phantom-limb pain: characteristics, causes, and treatment. Lancet Neurol.

[14] Maclver K, Lloyd DM, Kelly S, Roberts N, Nurmikko T (2008). Phantom limb pain, cortical reorganization and the therapeutic effect of mental imagery. Brain.

[15] Moseley GL, Sym DF, Henry ML, Souvalis T (2005). Experimental hand pain delays recognition of the contralateral hand-Evidence that acute and chronic pain have opposite effects on information processing?. Cogn Brain Res.

[16] Hodges PW, Moseley GL (2003). Pain and motor control of the lumbopelvic region: effect and possible mechanisms. J Electromyogr Kinesiol.

[17] Hodges PW, Moseley GL, Gabrielsson A, Gandevia SC (2003). Experimental muscle pain changes feedforward postural responses of the trunk muscles. Exp Brain Res.

[18] Luomajoki H, Kool J, De Bruin ED, Airaksinen O (2007). Reliability of movement control tests in the lumbar spine. BMC Musculoskelet Disord.

[19] Luomajoki H, Kool J, De Bruin ED, Airaksinen O (2008). Movement control tests of the low back; evaluation of the difference between patients with low back pain and healthy controls. BMC Musculoskelet Disord.

[20] Wand BM, O'Connell NE, Di Pietro F, Bulsara M (2011). Managing chronic non-specific low back pain with a sensorimotor retraining approach: exploratory multiple-baseline study of 3 participants. Phys Ther.

[21] Craig P, Dieppe P, Macintyre S, Michie S, Nazareth I, Petticrew M (2008). Developing and evaluating complex interventions: the new Medical Research Council guidance. BMJ.

[22] Roland MO, Morris RW (1983). A study of the natural history of back pain. Part 1: development of a reliable and sensitive measure of disability in low back pain. Spine.

[23] Wiesinger GF, Nuhr M, Michael Q, Ebenbichler G, Wölfl G, Fialka‐Moser V (1999). Cross‐cultural adaptation of the Roland‐Morris questionnaire for German‐speaking patients with low back pain. Spine.

[24] Hill JC, Whitehurst DG, Lewis M, Bryan S, Dunn KM, Foster NE (2011). Comparison of stratified primary care management for low back pain with current best practice (STarT Back): a randomised controlled trial. Lancet.

[25] Moseley LG, Butler DS (2012). Schmerzen Verstehen.

[26] Flor H, Birbaumer N (2000). Phantom limb pain: cortical plasticity and novel therapeutic approaches. Curr Opin Anaesthesiol.

[27] Luomajoki H, Moseley GL (2011). Tactile acuity and lumbopelvic motor control in patients with back pain and healthy controls. Br J Sports Med.

[28] Tsao H, Galea MP, Hodges PW (2010). Driving plasticity in the motor cortex in recurrent low back pain. Eur J Pain.

[29] Neuro Orthopaedic Institute. Recognise® software. 2011, 19 North St, Adelaide City West, South Australia 5000, Australia. [http://www.noigroup.com/Recognise/]

[30] Horn KK, Jennings S, Richardson G, Vliet DV, Hefford C, Abbott JH (2012). The patient-specific functional scale: psychometrics, clinimetrics, and application as a clinical outcome measure. J Orthop Sports Phys Ther.

[31] Waddell G, Newton M, Henderson I, Somerville D, Main CJ (1993). A Fear-Avoidance Beliefs Questionnaire (FABQ) and the role of fear-avoidance beliefs in chronic low back pain and disability. Pain.

[32] Staerkle R, Mannion AF, Elfering A, Junge A, Semmer NK, Jacobshagen N (2004). Longitudinal validation of the Fear-Avoidance Beliefs Questionnaire (FABQ) in a Swiss-German sample of low back pain patients. Eur Spine J.

[33] Sullivan MJL, Bishop SR, Pivik J (1995). The pain catastrophizing scale: development and validation. Psychol Assess.

[34] Meyer K, Sprott H, Mannion AF (2008). Cross-cultural adaptation, reliability, and validity of the German version of the Pain Catastrophizing Scale. J Psychosom Res.

[35] Catley MJ, Tabor A, Wand BM, Moseley GL (2013). Assessing tactile acuity in rheumatology and musculoskeletal medicine–how reliable are two-point discrimination tests at the neck, hand, back and foot?. Rheumatology (Oxford).

[36] Beinart NA, Goodchild CE, Weinman JA, Ayis S, Godfrey EL (2013). Individual and intervention-related factors associated with adherence to home exercise in chronic low back pain: a systematic review. Spine J.

[37] Rainville J, Ahern DK, Phalen L (1993). Altering beliefs about pain and impairment in a functionally oriented treatment program for chronic low back pain. Clin J Pain.

[38] Mannion AF, Balagué F, Pellisé F, Cedraschi C (2007). Pain measurement in patients with low back pain. Nat Clin Pract Rheumatol.

[39] Hägg O, Fritzell P, Nordwall A (2003). The clinical importance of changes in outcome scores after treatment for chronic low back pain. Eur Spine J.

[40] Dworkin RH, Turk DC, McDermott MP, Peirce-Sandner S, Burke LB, Cowan P (2009). Interpreting the clinical importance of group differences in chronic pain clinical trials: IMMPACT recommendations. Pain.

[41] Leon AC, Davis LL, Kraemer HC (2011). The role and interpretation of pilot studies in clinical research. J Psychiatr Res.

[42] Chapman JR, Norvell DC, Hermsmeyer JT, Bransford RJ, DeVine J, McGirt MJ (2011). Evaluating common outcomes for measuring treatment success for chronic low back pain. Spine.

[43] Faul F, Erdfelder E, Lang A-G, Buchner A (2007). G*Power 3: a flexible statistical power analysis program for the social, behavioral, and biomedical sciences. Behav Res Methods.

