# Supplementary material for: Short-term effect on pain and function of neurophysiological education and sensorimotor retraining compared to usual physiotherapy in patients with chronic or recurrent non-specific low back pain, a pilot randomized controlled trial
Source: BMC Musculoskelet Disord. 2015 Apr 10;16:83. doi: 10.1186/s12891-015-0533-2 (PMC4413527; doi:10.1186/s12891-015-0533-2)
Supplement: Additional file 1: — Content of sessions and home assignments of multimodal treatment. [file 12891_2015_533_MOESM1_ESM.pdf]

Additional file 1: Content of sessions and home assignments of multimodal treatment

Week	Content	Assignment (HTI* (5 assignments per week)
1	• personal login to the HTI<br>• instruction on HTI use for assignments<br>• patients receive the book Explain Pain<br>• neurophysiology of (chronic) back pain<br>• local trunk muscle recruitment, dissociation exercises, movement control	• reading section of the book Explain Pain<br>• answer 18 questions related to the section read<br>• movement control exercise, frequency and duration reported in HTI
2	• neurophysiology of pain<br>• movement control exercises<br>• instruction on left-right recognition training with the Recognise® program	• similar to previous week<br>• left-right recognition training with 3 x 20 pictures of trunk positions
3	• instruction on SRT<br>• neurophysiology of pain and analysis of patient's individual situation<br>• purpose of SRLM explained with video<br>• movement control exercises	• perform SRT on HTI (3 x 40 two numbered dots to discern)<br>• watching video with SRLM (linked on HTI)<br>• movement control exercise, frequency and duration reported in HTI
4	• education on the application of movement control into daily living<br>• graded exposure to functional movement	• perform SRT on HTI (3 x 40 numbered dots)<br>• perform 3 x 20 images on Recognise®<br>• movement control exercise, frequency and duration reported in HTI
5	• analysis of everyday movements, discussion of experienced limitations, finding better movement strategies	• perform SRT on HTI (1 x 40 letters to perceive)<br>• watching video with SRLM<br>• movement control exercise, frequency and duration reported in HTI
6	• as in previous session<br>• emphasis on individual resources and recreation	• perform SRT on HTI (1 x 30 3-letter-words to perceive)<br>• watching video with FRLM<br>• movement control exercise, frequency and duration reported in HTI
7	• as in previous session	• perform SRT on HTI (3 x 40 numbered dots)<br>• watching video with FRLM<br>• movement control exercise, frequency and duration reported in HTI
8	• wrap-up of acquired knowledge, newly integrated movement behaviour and handling of pain exacerbations in the future	• reading a summary of the key points of the programme, including application to everyday life<br>• watching video with FRLM<br>• movement control exercise, frequency and duration reported in HTI

HTI web-based home training interface; SRT Sensory Retraining Tool (discerning numbered dots, pressed on patient's back); SRLM small range lumbar movement (to observe and perform, such as in a gymnastic tutorial video); FRLM full range lumbar movement.
