# Supplementary material for: Short-term effect on pain and function of neurophysiological education and sensorimotor retraining compared to usual physiotherapy in patients with chronic or recurrent non-specific low back pain, a pilot randomized controlled trial
Source: BMC Musculoskelet Disord. 2015 Apr 10;16:83. doi: 10.1186/s12891-015-0533-2 (PMC4413527; doi:10.1186/s12891-015-0533-2)
Supplement: Additional file 2: — Recruitment sources. [file 12891_2015_533_MOESM2_ESM.pdf]

**Additional file 2: Recruitment sources**

| | |
|---|---|
| Regularly referred to the study practice | 9 |
| Mailing to employees by company | 9 |
| Newspaper advertisement | 5 |
| University sports website | 3 |
| Flyer on notice board in supermarket and fitness centre | 2 |
