# Supplementary material for: Short-term effect on pain and function of neurophysiological education and sensorimotor retraining compared to usual physiotherapy in patients with chronic or recurrent non-specific low back pain, a pilot randomized controlled trial
Source: BMC Musculoskelet Disord. 2015 Apr 10;16:83. doi: 10.1186/s12891-015-0533-2 (PMC4413527; doi:10.1186/s12891-015-0533-2)
Supplement: Additional file 3: — Online eligibility testing. [file 12891_2015_533_MOESM3_ESM.pdf]

Additional file 3: Online eligibility testing

Persons contacting online recruitment homepage: 372	Reasons for exclusion (-n)
Paralysis on one or both lower limbs	-60
Not having an aid that helps with home assignments over 5 weeks, 15 minutes, five times a week	-41
Suffering from LBP less than 3 months	-19
Intra-articular or perineural steroid injection on the lumbar spine during the previous five months	-17
Surgery on the lumbar spine within the last 2 years	-13
Age < 18 or > 60	-11
History of fracture of the lumbar spine	-4
Pregnancy or >5 months postpartum	-3
Not capable of reading and speaking German	-3
Contraindication to physical exercise	-2
No home access to the internet	-1
Less than 5 points on the RMDQ	-84
Less than 4 points on the KSBT	-78
Not inclined to participate after detailed explication about the program	-8
	28 included after signing informed consent
