# Supplementary material for: Short-term effect on pain and function of neurophysiological education and sensorimotor retraining compared to usual physiotherapy in patients with chronic or recurrent non-specific low back pain, a pilot randomized controlled trial
Source: BMC Musculoskelet Disord. 2015 Apr 10;16:83. doi: 10.1186/s12891-015-0533-2 (PMC4413527; doi:10.1186/s12891-015-0533-2)
Supplement: Additional file 4: — MMT Patients satisfaction with treatment. [file 12891_2015_533_MOESM4_ESM.pdf]

Additional file 4: MMT Patients satisfaction with treatment

Frequency of answers (total 14)

**Achieved outcome**	1 strongly agree	2 agree	3 neither agree nor disagree	4 disagree	5 strongly disagree	Missing data
I'm satisfied with the achieved outcome	7	4<br>1 wd/t<br>1 wd/c<br>1 wd/i				
I reached the conjointly pre-assigned aims	8	3<br>1 wd/t<br>1 wd/c				1 m/i
**Neurophysiological education**						
The length of the chapters to read in the book Explain Pain was o.k. for me	8	3<br>1 wd/t<br>1 wd/c	1 wd/i			
The content of the chapters to read in the book Explain Pain was comprehensible for me	6	4<br>1 wd/i	1<br>1 wd/t<br>1 wd/c			
The amount of questions to answer according to the read chapters was o.k. for me	6	3<br>1 wd/t<br>1 wd/c	2	1 wd/i		
The level of difficulty of the questions to answer according to the read chapters was o.k. for me	5	4<br>1 wd/c	2<br>1 wd/t	1 wd/i		
The appliance of the home training interface (HTI) (what chapter to read, how to fill in the questions) was comprehensible for me	8<br>1 wd/t	2<br>1 wd/c	2	1 wd/i		
The content of the therapy sessions according to neurophysiology education was comprehensible for me	7<br>1 wd/t	3<br>1 wd/c	1			1 wd/i
The content of the written chapters in the book Explain Pain and the relevant questions were helpful	5	5<br>1 wd/c	1<br>1 wd/t			1 wd/i
The content of the therapy sessions according to neurophysiology education was helpful	7	4<br>1 wd/c	1 wd/t			1 wd/i
**Sensory retraining with Sensory Retraining Tool (SRT), discerning numbered dots**						
The expenditure of time to execute the SRT was o.k. for me	3	7<br>1 wd/c	1<br>1 wd/i		1 wd/t	
The expenditure of time to execute the SRT was o.k. for my aid person	2	4	3<br>1 wd/c<br>1 wd/i	1	1 wd/t	
The procedure how to apply the SRT was comprehensible for me	6<br>1 wd/t	5<br>1 wd/c		1 wd/i		

The whole purpose of the SRT was comprehensible for me	7<br>1 wd/t	4<br>1 wd/c<br>1 wd/i				
The execution of the SRT did help me	4	6<br>1 wd/c<br>1 wd/i	1<br>1 wd/t			
**Sensory retraining with Sensory Retraining Tool (SRT), single letters and three-letter-words**						
The expenditure of time to execute the SRT was o.k. for me	5	5	1<br>1 wd/c			1 wd/t<br>1 wd/i
The expenditure of time to execute the SRT was o.k. for my aid person	4	2	4<br>1 wd/c	1		1 wd/t<br>1 wd/i
The procedure how to apply the SRT was comprehensible for me	9	2<br>1 wd/c				1 wd/t<br>1 wd/i
The whole purpose of the SRT was comprehensible for me	9	2<br>1 wd/c				1 wd/t<br>1 wd/i
The execution of the SRT did help me	5	5<br>1 wd/c	1			1 wd/t<br>1 wd/i
**Determination of perspective of pictures of the trunk on Recognise® Software**						
The preceding information on how to apply Recognise® was comprehensible for me	9<br>1 wd/t	2<br>1 wd/c<br>1 wd/i				
The expenditure of time to execute the Recognise® home training was o.k. for me	8<br>1 wd/t	2	1<br>1 wd/c 1 wd/i			
The whole purpose of the Recognise® home training was comprehensible for me	8<br>1 wd/t	3<br>1 wd/c<br>1 wd/i				
The Recognise® home training did help me	4	6<br>1 wd/t<br>1 wd/c	1<br>1 wd/i			
**Video small range lumbar movements (SRLM)**						
The preceding information, what to do when watching the SLRM-video at home was comprehensible for me	8<br>1 wd/t	2<br>1 wd/c<br>1 wd/i	1			
**Video full range lumbar movements (FRLM)**						
The preceding information, what to do when watching the FRLM-video at home was comprehensible for me	8	2<br>1wd/c	1			1 wd/t<br>1 wd/i
**Open questions**						
What helped me most	‘intentional change of posture’; ‘improvement of body-awareness’; ‘reading the book, therapist who showed me motor control exercises and supervised me’; ‘motor control exercises and sensory retraining 1 wd/t’; ‘the newly acquired knowledge that pain is no physical sensation but triggered from within the brain’; ‘combination of SRT with aid and knowledge from the book’; ‘the book and motor control exercises 1 wd/c’; ‘reading the					1 wd/t

	book, therapy sessions'; 'explanations of therapist, supervision of motor control exercises to change bad movement patterns'; 'knowledge about conduction of pain'; 'motor control exercises to change bad movement patterns, learning selective movement'; 'reading the book'; 'performing motor control exercises regularly, for example how to get out of my car without pain';	
What helped me least	'don't know, eventually the SRT'; 'recognise'; ' video S/FRLM'; 'reading the book 1 wd/t'; 'eventually watching the video'; 'SRT 1 wd/c'; 'SRT'; 'video S/FRLM'; 'recognise'; 'all together helped, the combination was the key'; 'SRT';	1 wd/i
What I liked least	'SRT'; 'executing the 3rd time in a row the STR (numbers)'; 'SRT numbers'; 'reading the book and answering questions 1 wd/t'; 'the SRT numbers'; 'to persuade my aid to help me for the SRT'; 'SRT 1 wd/c'; 'SRT'; 'video S/FRLM'; SRT'; 'motor control exercises'; 'SRT letters'; 'nothing, maybe the lengthy stories in the book';	1 wd/i
What I liked the most	'recognise'; 'active exercises for motor retraining'; 'SRT letters, motor control exercises, reading the book'; 'video SRLM and performing exercises simultaneously 1 wd/t'; 'the SRT letters'; 'the diversity and alternation of interventions'; 'motor control exercises 1 wd/c'; 'reading the book'; 'motor control exercises at home'; 'motor control exercises'; 'recognise'; 'motor control exercises'; 'practising motor control movements during the physiotherapy sessions'	1 do/i

1 wd/t: one early withdrawal after 7th week: aid person couldn't afford time to help the subject to execute the SRT. 1 wd/c: one early withdrawal after 4th week because inappropriate and misleading use of the home training interface. 1 wd/i: one early withdrawal after the 6th week because of software problems with the home training to run on android system
