# Supplementary material for: Short-term effect on pain and function of neurophysiological education and sensorimotor retraining compared to usual physiotherapy in patients with chronic or recurrent non-specific low back pain, a pilot randomized controlled trial
Source: BMC Musculoskelet Disord. 2015 Apr 10;16:83. doi: 10.1186/s12891-015-0533-2 (PMC4413527; doi:10.1186/s12891-015-0533-2)
Supplement: Additional file 5: — UPT Patients satisfaction with treatment. [file 12891_2015_533_MOESM5_ESM.pdf]

Additional file 5: UPT Patients satisfaction with treatment

	Frequency of answers (total 13)				
	1 strongly agree	2 agree	3 neither agree nor disagree	4 disagree	5 strongly disagree
**Achieved outcome**					
I'm satisfied with the achieved outcome	1	7<br>1 wd/t	2	1	1 wd/e
I reached the conjointly pre-assigned aims	1	6<br>1 wd/t	2	2	1 wd/e
**Home training and usage of the home training interface (HTI)**					
The explanations for the usage of home training interface were comprehensible. When I was at home I knew exactly how to operate the HTI	7 (1 do/t) (1 do/e)	3	1		
The selection of exercises was o.k. for me	6	4<br>1 wd/t<br>1 wd/e	1		
I could follow the whole purpose of the exercises I was given	5<br>1 wd/t	6<br>1 wd/e			
The expenditure of time for the home training and reporting on the HTI was o.k. for me	3	6<br>1 wd/t<br>1 wd/e	2		
The exact reporting on the HTI, how often and to what expenditure of time I had done the exercises was o.k. for me	1	5	3<br>1 wd/t<br>1 wd/e	2	
The regular performance of the home training was helpful	4	6<br>1 wd/t	1		1 wd/e
**Therapy sessions**					
I was satisfied with the content of the therapy sessions	5<br>1 wd/t	6<br>1 wd/e			
The frequency of the therapy sessions was too low for me	1<br>1 wd/e		1	2	7<br>1 wd/t
The frequency of the therapy sessions was totally fine for me	7<br>1 wd/t	3	1 wd/e		1
The content of the therapy sessions was helpful	4	7<br>1 wd/t			1 wd/e
**Open questions**					

What helped me the most	'the treatments on my back (massage etc.)'; 'the exercises and the therapy sessions'; 'regular execution of exercises and sport-tape'; 'regular execution of exercises'; 'my partner'; 'the pain got worse (wd/e); 'that I started to work out again'; 'the motivation of the therapist'; 'expansion of my exercise-repertoire'; 'alternated exercises'; 'stretching exercises, tips how to perform strengthening exercises'; 'many tips for different exercises'; 'input from therapist';
What helped me least	'?' 4x; coming too late to the therapy sessions'; 'the pain got worse (wd/e)'; 'nothing' 5x; 'background of the study not transparent'; 'when therapist was on holiday one week';

What I liked least	‘to take the time to perform the training program’; ‘scarf-therapy’; ‘strengthening exercises’ 2x; ‘helpful was the massage (do/e)’; ‘the reporting on the HTI 2x’; ‘the aching of my back’; ‘the regular performance of exercises 2x’; ‘the reporting on the HTI’; ‘massage, triggerpoint treatment trough belly region’; ‘to report on the HTI’
What I liked the most	‘Exercises for back and trunk 2x’; ‘the therapy sessions’; ‘exercises’; ‘exercises for relaxation and stretching’; ‘the advice from the therapist (do/e)’; ‘the regular execution of exercises’; ‘the therapists healing methods’; ‘the massage 2x’; ‘the massage’; ‘treatment was good, but nothing to like ☺’; ‘to learn more about my body’;

1 wd/t: early withdrawal after 2nd week: couldn’t afford time to execute home training. 1 wd/e: early withdrawal after 3rd week: because of exacerbation of LBP
